# Comparative Analyses of Complete Peronosporaceae (Oomycota) Mitogenome Sequences—Insights into Structural Evolution and Phylogeny

**DOI:** 10.1093/gbe/evac049

**Published:** 2022-04-14

**Authors:** Richard C. Winkworth, Grace Neal, Raeya A. Ogas, Briana C. W. Nelson, Patricia A. McLenachan, Stanley E. Bellgard, Peter J. Lockhart

**Affiliations:** 1 Bio-Protection Research Centre, Massey University, Palmerston North, New Zealand; 2 School of Natural Sciences, Massey University, Palmerston North, New Zealand

**Keywords:** genome rearrangements, inverted repeats, mitochondrial genome, Peronosporales, sequence evolution, structural diversity, synteny

## Abstract

Members of the Peronosporaceae (Oomycota, Chromista), which currently consists of 25 genera and approximately 1,000 recognized species, are responsible for disease on a wide range of plant hosts. Molecular phylogenetic analyses over the last two decades have improved our understanding of evolutionary relationships within Peronosporaceae. To date, 16 numbered and three named clades have been recognized; it is clear from these studies that the current taxonomy does not reflect evolutionary relationships. Whole organelle genome sequences are an increasingly important source of phylogenetic information, and in this study, we present comparative and phylogenetic analyses of mitogenome sequences from 15 of the 19 currently recognized clades of Peronosporaceae, including 44 newly assembled sequences. Our analyses suggest strong conservation of mitogenome size and gene content across Peronosporaceae but, as previous studies have suggested, limited conservation of synteny. Specifically, we identified 28 distinct syntenies amongst the 71 examined isolates. Moreover, 19 of the isolates contained inverted or direct repeats, suggesting repeated sequences may be more common than previously thought. In terms of phylogenetic relationships, our analyses of 34 concatenated mitochondrial gene sequences resulted in a topology that was broadly consistent with previous studies. However, unlike previous studies concatenated mitochondrial sequences provided strong support for higher-level relationships within the family.

**Significance**

Mitochondrial sequences are commonly used for distinguishing amongst species and reconstructing evolutionary relationships. However, for many lineages, mitochondrial genomes are poorly characterized. We explored gene content, gene order, and evolutionary relationships using 44 newly assembled and 27 publicly available mitogenome sequences for Peronosporaceae. Our results provide new insights into genome and lineage evolution in this economically important group of plant pathogens.

## Introduction

The Peronosporaceae (Oomycota, Chromista) is a monophyletic group of biotrophic and hemibiotrophic phytopathogens (Thines and Choi 2016). Most of the approximately 1,000 species currently placed in the family belong to either *Peronospora* (∼500 species) or *Phytophthora* (∼200 species) with the remainder divided amongst 23 smaller genera including *Bremia*, *Hyaloperonospora*, and *Plasmopara* (Thines 2014; Thines and Choi 2016). As a group, the Peronosporaceae infect a wide array of host plants, impacting both agricultural and natural ecosystems (e.g., Hansen et al. 2012). Well-known diseases attributed to members of the family include lettuce downy mildew (*Bremia lactucae*), grape downy mildew (*Plasmopara viticola*) potato late blight (*Phytophthora infestans*), and sudden oak death (*Phytophthora ramorum*) (Thines 2014; Thines and Choi 2016).

**Fig. 1.**
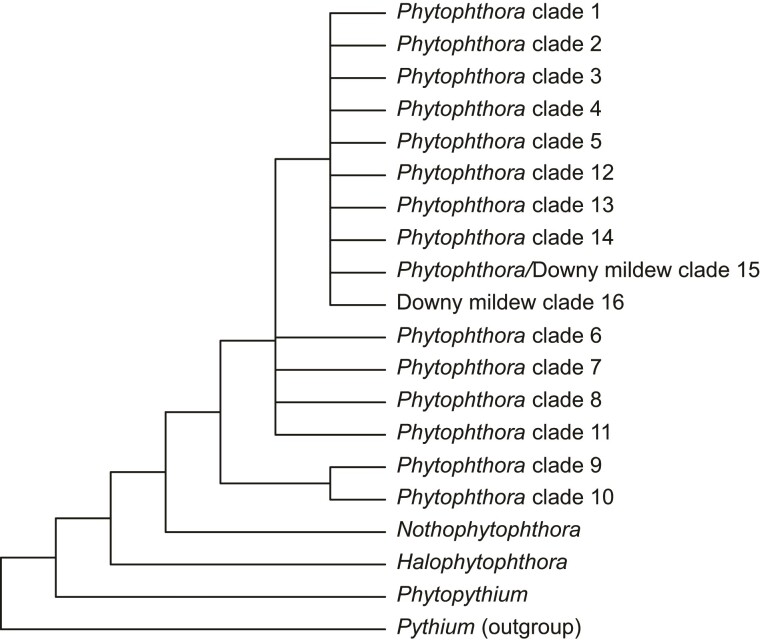
Schematic diagram summarizing our current understanding of evolutionary relationships within Peronosporaceae. Included are 14 numbered clades comprised exclusively of *Phytophthora*, two numbered clades comprised exclusively (Clade 16) or almost so (Clade 15) of representatives of the 21 downy mildew genera and three early-diverging clades broadly corresponding to *Halophytophthora*, *Nothophytophthora*, and *Phytopythium*. The cladogram is based on the results of Jung et al. (2017a, 2017b), Yang et al. (2017), and Bourret et al. (2018).

Our understanding of evolutionary relationships within Peronosporaceae has improved dramatically over the last two decades. To date much of the focus has been on *Phytophthora* (e.g., Cooke et al. 2000, 2002; Kroon et al. 2004; Blair et al. 2008; Martin et al. 2014). In their study, Cooke et al. (2000) analysed nuclear ribosomal internal transcribed spacer sequences from 47 *Phytophthora* species and identified 10 clades. Subsequent studies (e.g., Blair et al. 2008; Martin et al. 2014) have included larger samples of both taxa and molecular loci. In addition to providing further support for the groups identified by Cooke et al. (2000), these studies have identified additional clades (e.g., Jung et al. 2017a; Bourret et al. 2018). For example, Bourret et al. (2018) included 135 taxa representing 22 genera of the Peronosporaceae and recognized 16 numbered clades, only one of which did not contain a member of *Phytophthora* (fig. 1). In addition to numbered clades, three named lineages have been recognized. Specifically, clades corresponding to *Halophytophthora*, *Nothphytophthora*, and *Phytopythium* have been identified as early-diverging within Peronosporaceae (Jung et al. 2017b; fig. 1). Despite an improved understanding of the major groupings within Peronosporaceae, relationships among them have remained uncertain. For example, the analyses of Blair et al. (2008), Martin et al. (2014), and Bourret et al. (2018) do not consistently resolve relationships amongst the numbered clades.

Our understanding of species diversity within Peronosporaceae has also dramatically improved over the last two decades. In *Phytophthora* alone, the number of formally recognized taxa has grown from 60 in 1996 (Erwin and Ribeiro 1996) to in excess of 150 (Thines and Choi 2016; Yang et al. 2017). For example, Yang et al. (2017) included 142 formally described and 43 yet to be described *Phytophthora* entities in their phylogenetic analyses. Rapid growth in the size of the family reflects increased attention on understanding the diversity in natural ecosystems such as forests (e.g., Vettraino et al. 2002; Jung et al. 2017a) and streams (e.g., Reeser et al. 2007; Yang et al. 2016; Brazee et al. 2017) as well as in regions such as Asia and South America (e.g., Webber et al. 2011; Lee et al. 2017; Jung et al. 2020; Legeay et al. 2020).

Mitochondria maintain a circular, extra-nuclear genome. In metazoans, this genome is typically uniparentally inherited, structurally conserved, and evolves rapidly at the nucleotide sequence level. As a consequence, the mitochondrial genome has become an important source of sequence data for resolving evolutionary relationships in animals (Curole and Kocher 1999; Sullivan et al. 2017). In contrast, the mitogenomes of many other groups are more poorly characterized. At least in part, this is due to marked differences in the size and complexity of mitogenomes within and between groups. For example, fungal mitogenomes vary from approximately 20 kb to more than 235 kb in size (Sandor et al. 2018).

Complete mitogenome sequences are available for species representing eight oomycete genera. Specifically, sequences are available for *Achlya* (O’Brien et al. 2014), *Bremia*, *Peronospora* (Derevnina et al. 2015; Fletcher et al. 2018), *Phytophthora* (e.g., Avila-Adame et al. 2006; Martin et al. 2007; Lassiter et al. 2015; Cai and Scofield 2020; McGowan et al. 2020), *Pseudoperonospora* (Rahman et al. 2019), *Pythium* (Lévesque et al. 2010; Tangphatsornruang et al. 2016), *Saprolegnia* (Grayburn et al. 2004), and *Thraustotheca* (O’Brien et al. 2014). In most cases, mitochondrial genome sequences have been reported for just one (e.g., *Bremia*, *Saprolegnia*) or two (e.g, *Pseudoperonospora*) taxa. However, sequences have been reported for 17 members of *Phytophthora*. Currently, nine of the numbered clades recognized by Bourret et al. (2018) are represented by one or more complete mitogenome sequences. Comparative analyses of the available *Phytophthora* mitochondrial genome sequences have indicated that despite broad conservation of gene content, gene order varies both within and between taxa (e.g., Martin et al. 2007; Yuan et al. 2017; McGowan et al. 2020). Such analyses also suggest that unlike the mitogenomes of Pythiaceae and Saprolegniaceae, those of *Phytophthora* typically lack inverted repeats.

In this study, we present comparative and phylogenetic analyses of mitogenomes from a broad sample of Peronosporaceae. We include 44 newly assembled mitochondrial genome sequences representing 34 members of Peronosporaceae; for all but one of these taxa mitogenome sequences have not previously been reported. Overall, our sample includes 15 of the 19 currently recognized clades of Peronosporaceae and, based on Yang et al. (2017), approximately 25% (45 taxa) of recognized *Phytophthora* diversity. We discuss our results with respect to the evolution of mitochondrial genome diversity in Peronosporaceae.

## Results

We assembled complete mitochondrial genome sequences from 44 representatives of the Peronosporaceae. Mitogenomes were considered complete when the ends of linear drafts were circularized, and no inconsistencies (e.g., gaps or misalignments) were observed when the original sequence reads were mapped to the draft. Average read coverage for the 44 mitogenomes ranged from 528.8 to 38,991.5 reads (table 1).

**Table 1:** Summary Statistics for the Mitochondrial Genome Sequences of the Included Peronosporaceae

Species	Genome		Proportion of genome		Gene numbers		
	Size	Mean read depth	Coding	Noncoding	Protein	rRNA	tRNA
Clade 1							
*Phytophthora aleatoria*	38,936	4,417.2	0.88	0.12	39	2	25
*Phytophthora andina*	37,874	–	0.9	0.1	38	2	25
*Phytophthora cactorum*	38,068	23,066	0.9	0.1	39	2	25
*Phytophthora infestans*	37,922, 37,957, 39,840, 39,870	–	0.86–0.90	0.10–0.14	39	2	25
*Phytophthora ipomoeae*	37,872	–	0.9	0.1	39	2	25
*Phytophthora mirabilis*	37,779	–	0.9	0.1	38	2	25
*Phytophthora nicotianae*	37,673, 37,749	4,180.1–14,772.4	0.90–0.91	0.09–0.10	39	2	25
*Phytophthora phaseoli*	37,914	–	0.9	0.1	38	2	25
Clade 2							
*Phytophthora capsici*	38,418, 38,427	5,258.3–10,992.9	0.89	0.11	39	2	25
*Phytophthora colocasiae*.	41,297, 41,367	7,474.4–12,996.5	0.82–0.83	0.17–0.18	39	2	25
*Phytophthora multivora*	37,992, 38,032	1,016.6–1,724.3	0.9	0.1	39	2	25
*Phytophthora plurivora*	38,082	7,334.1	0.9	0.1	39	2	25
*Phytophthora* sp. subnubulis	37,854	10,810.8	0.9	0.1	39	2	25
*Phytophthora tropicalis*	37,047	8,908.8	0.92	0.08	39	2	25
Clade 3							
*Phytophthora pseudosyringae*	39,143	–	0.87	0.13	39	2	25
*Phytophthora pluvialis*	39,325, 39,327	2,163.9–2,175.2	0.87	0.13	39	2	25
Clade 4							
*Phytophthora litchii*	37,950	940.8	0.9	0.1	39	2	25
*Phytophthora megakarya*	39,277	6,648	0.87	0.13	39	2	25
*Phytophthora palmivora*	38,741, 40,799	1,081.6–3,269.6	0.84–0.88	0.12–0.16	39	2	25
Clade 5							
*Phytophthora agathidicida*	36,826, 36,844	2,492.0–4,458.4	0.93	0.07	39	2	25
*Phytophthora castaneae*	37,083	3,535.3	0.92	0.08	39	2	25
*Phytophthora cocois*	37,078, 37,125	1,904.2–6,857.7	0.92	0.08	39	2	25
*Phytophthora heveae*	37,150	4,165.4	0.92	0.08	39	2	25
*Phytophthora* sp. novaeguineae	37,072	5,056	0.92	0.08	39	2	25
Clade 6							
*Phytophthora chlamydospora*	38,329	4,694.7	0.89	0.11	39	2	25
*Phytophthora gonapodyides*	43,974	–	0.78	0.22	39	2	25
*Phytophthora pinifolia*	43,061	19,135.3	0.88	0.12	43	2	27
Clade 7							
*Phytophthora* × *alni*	45,343	8,717.9	0.77	0.23	40	2	25
*Phytophthora* × *cambivora*	51,184	6,800.8	0.67	0.33	39	2	25
*Phytophthora cinnamomi*	39,225, 39,230	923.6–8,044.3	0.87	0.13	39	2	25
*Phytophthora fragariae*	44,706	3,548.9	0.76	0.24	39	2	25
*Phytophthora rubi*	45,523	7,993.4	0.76	0.24	40	2	25
*Phytophthora sojae*	42,977	–	0.8	0.2	40	2	25
Clade 8							
*Phytophthora cryptogea*	38,163	6,829.4	0.89	0.11	39	2	25
*Phytophthora lateralis*	38,507	5,606.1–38,991.5	0.89	0.11	39	2	26
*Phytophthora ramorum*	39,314, 39,494	–	0.87–0.88	0.12–0.13	40	2	26
*Phytophthora sansomeana*	39,618	–	0.87	0.13	40	2	25
Clade 9							
*Phytophthora capitosa*	44,662	3,126	0.76	0.24	39	2	25
*Phytophthora fallax*	43,681	5,049	0.78	0.22	39	2	25
*Phytophthora polonica*	40,467	–	0.84	0.16	39	2	25
Clade 10							
*Phytophthora kernoviae*	37,467	528.8–1,357.8	0.91	0.09	39	2	27
Clade 12							
*Phytophthora quercina*	38,471	10,559.3	0.89	0.11	39	2	25
*Phytophthora tubulina*	38,065	4,667	0.9	0.1	39	2	25
*Phytophthora versiformis*	38,011	2,937.7	0.9	0.1	39	2	25
Clade 15							
*Hyaloperonospora arabidopsidis*	38,797	3,648.9	0.88	0.12	39	2	25
*Peronospora belbahrii*	40,063	5,035	0.85	0.15	39	2	25
*Peronospora effusa*	41,318	–	0.83	0.17	39	2	25
*Peronospora tabacina*	43,225	–	0.79	0.21	39	2	25
*Phytophthora podocarpi*	38,130, 38,136	1,934.9–3,009.7	0.9	0.1	39	2	26
*Pseudoperonospora humuli*	39,087	–	0.87	0.13	39	2	25
Clade 16							
*Bremia lactucae*	39,302	–	0.87	0.13	39	2	26
*Plasmopara halstedii*	38,944	6,619.1	0.88	0.12	39	2	27
*Nothophytophthora*							
*Nothophytophthora* sp.	38,518	6,444.8	0.89	0.11	39	2	27
*Phytopythium*							
*Phytopythium vexans*	61,242	5,231.9	0.91	0.09	65	4	42
Pythiaceae							
*Pythium ultimum* Trow	59,689	–	0.89	0.11	63	4	42

### Genome Size

The 71 complete Peronosporaceae mitochondrial genomes ranged in size from 36,826 base pair (bp) for *Phytophthora agathidicida* to 61,242 bp for *Phytopythium vexans*. However, 76.1% (56/71) were <40,000 bp in length and only 5.6% (4/71) were >45,000 bp (table 1). A Wilcoxon rank-sum test comparing mitogenome lengths for our sample to publicly available sequences for other oomycetes (e.g., Lévesque et al. 2010; O’Brien et al. 2014; Tangphatsornruang et al. 2016) indicated that those of the Peronosporaceae were significantly smaller (*n*_Peronosporaceae_ = 71, *n*_oomycetes_ = 8, *W* = 13, *P* = 0.000012).

Large (i.e., 7,772–21,950 bp) inverted repeats are a dominant feature of publicly available sequences for other oomycetes (e.g., Lévesque et al. 2010; O’Brien et al. 2014; Tangphatsornruang et al. 2016). The mitogenomes of 25.5% (15/55) of the sampled taxa contained inverted repeats, including representatives of *Peronospora*, *Phytophthora*, *Plasmopara*, and *Phytopythium.* Direct repeats were identified in the *B. lactucae*, *P. polonica*, *P. sansomeana*, *P. sojae*, and *Pe. tabacina* mitogenomes. With just one exception repeated sequences in Peronosporaceae mitogenomes were shorter and their contribution to the overall size of the mitogenome was less than for the other oomycetes. Specifically, in Peronosporaceae the repeats were >5,442 bp in length and represented 1.2–21.3% of the corresponding genome length. The exception was *Ph. vexans*. In this case, the inverted repeats were 24,191 bp in length and accounted for 79.0% of the overall genome length.

Our sample of mitogenome sequences contained 14 taxa represented by sequences from two isolates and for *P. infestans* we included sequences representing each of the four haplotypes described by Avila-Adame et al. (2006). For three taxa—*P. chlamydospora*, *P. kernoviae*, and *P. lateralis*—both mitogenome sequences were the same length. Differences in mitogenome length were <200 bp for a further 10 taxa (e.g., *P. agathidicida*, *P. capsici*, and *P. multivora*) but for amongst isolates of *P. infestans* and *P. palmivora* were 1,948 and 2,058 bp, respectively (table 1).

### Gene Content and Order

Coding sequences comprised 66.8–92.7% of the sampled Peronosporaceae mitogenomes; protein coding, tRNA, and rRNA genes accounted for 55.0–76.3%, 3.7–5.4%, and 8.1–14.0%, respectively. The remaining 7.3–33.2% of these sequences were noncoding. No introns were identified, in all cases the entire noncoding component was intergenic.

**Fig. 2.**
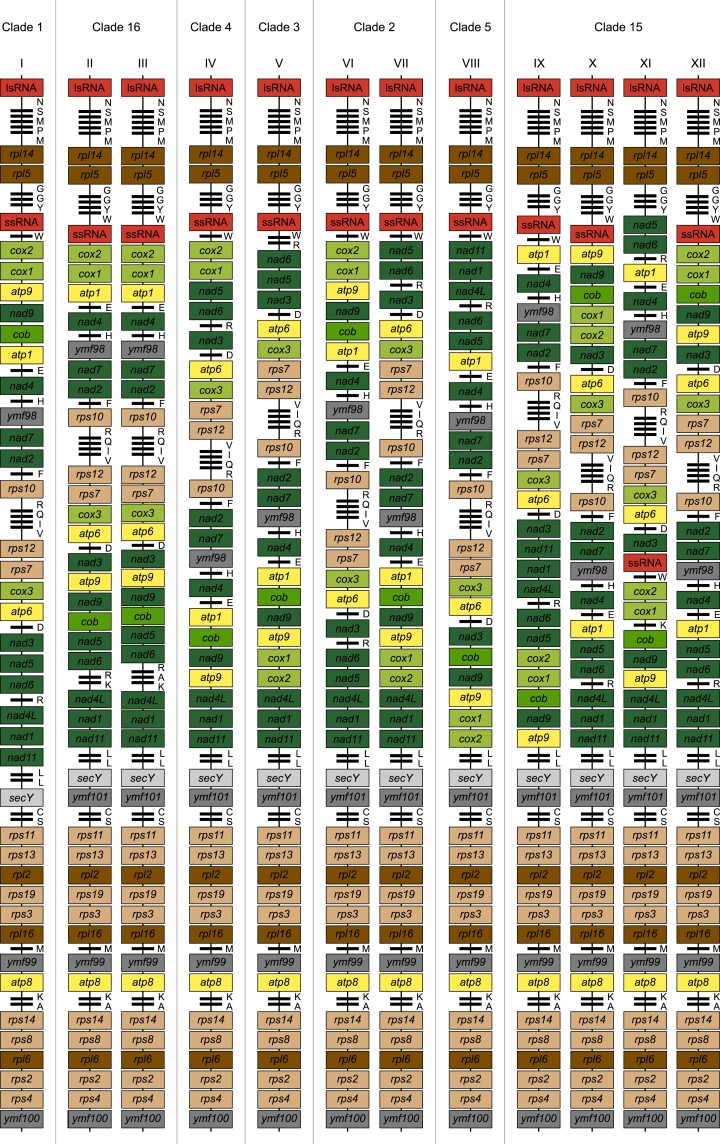

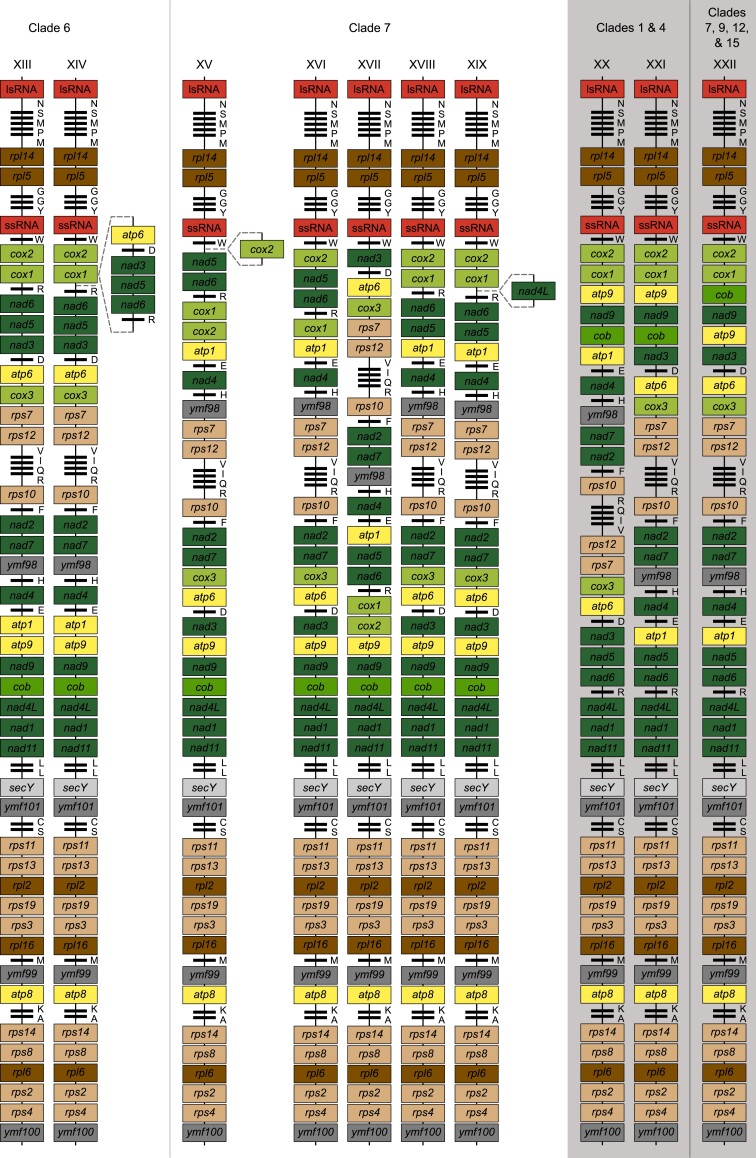

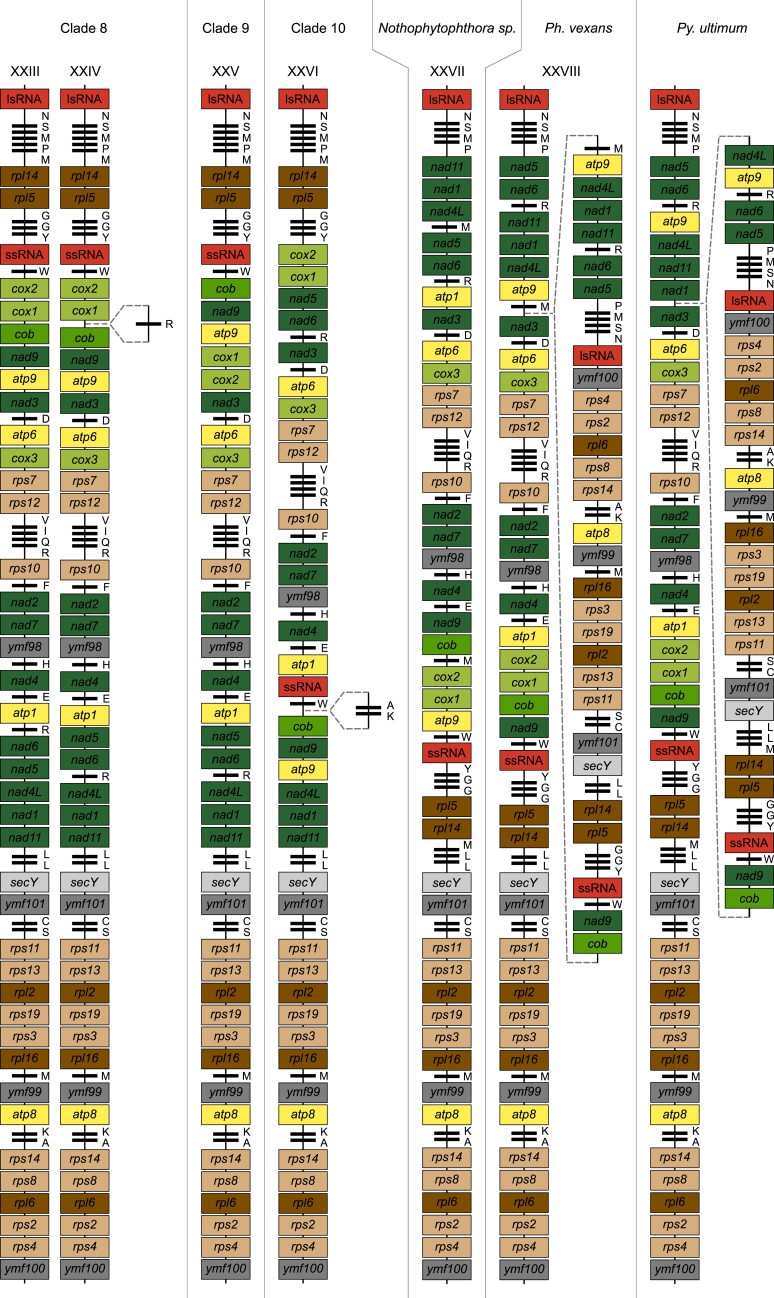
Schematic diagrams of the 28 mitochondrial syntenies identified recovered for Peronosporaceae plus that of *Pythium ultimum* (Pythiaceae). Genomes are oriented relative to the large ribosomal subunit with protein encoding and rRNA genes represented by colored rectangles labeled with standard gene abbreviations and tRNA genes represented by thick black lines labeled with the one-letter code for the corresponding amino acid. Different syntenies are labeled with Roman numerals I, *P. andina*, *P. ipomoeae*, *P. mirabilis*, and *P. phaseoli*; II, *B. lactucae*; III, *Pl. halstedii*; IV, *P. megakarya*; V, *P. pseudosyringae* and *P. pluvialis*; VI, *P. capsici*, *P. multivora*, *P. plurivora*, and *P. tropicalis*; VII, *P. colocasiae* and *P.* sp. subnubulis; VIII, *P. agathidicida*, *P. castaneae*, *P. cocois*, *P. heveae*, and *P.* sp. novaeguineae; IX, *H. arabidopsidis*; X, *Pe. belbahrii*; XI, *P. podocarpi*; XII, *Ps. humuli*; XIII, *P. chlamydospora* and *P. gonapodyides*; XIV, *P. pinifolia*; XV, *P. alni*; XVI, *P. cambivora*; XVII, *P. cinnamomi*; XVIII, *P. fragariae*; XIX, *P. rubi*; XX, *P. infestans* and *P. palmivora*; XXI, *P. aleatoria*, *P. cactorum*, *P. nicotianae*, and *P. litchii*; XXII, *Pe. effusa*, *Pe. tabacina*, *P. polonica*, *P. quercina*, *P. sojae*, *P. tubulina*, and *P.versiformis*; XXIII, *P. cryptogea* and *P. sansomeana*; XXIV, *P. lateralis* and *P. ramorum*; XXV, *P. captiosa* and *P. fallax*; XXVI, *P. kernoviae*; XXVII, *Nothophytophthora* sp.; XXVIII, *Ph. vexans*. Broadly, genomes are ordered based on the arrangement of clades in Fig. 3.

For 66.2% (47/71) of the sampled Peronosporaceae mitogenomes, the gene content included 39 protein encoding, 2 rRNA, and 25 tRNA genes. A further 15.5% (11/71) had 39 protein encoding and 2 rRNA genes but 26–27 tRNA genes. All but one of the remaining Peronosporaceae mitogenomes (16.9%, 12/71) had 38, 40, or 43 protein encoding, 2 rRNA, and 25–27 tRNA genes (fig. 2). The exception was the *Ph. vexans* mitogenome, which contained 65 protein encoding, 4 rRNA, and 42 tRNA genes. Differences in gene content were associated with inverted repeats (e.g., *Ph. vexans* and *P. alni*) or with a reduced number of hypothetical protein genes (i.e., *ymf98*, *ymf99*, *ymf100*, and *ymf101*) identified. Specifically, mitogenomes containing 39 protein-encoding genes typically had four *ymf* genes whereas the closely related clade 1 species *P. andina*, *P. ipomoeae*, *P. mirabilis*, and *P. phaseoli* all lacked the *ymf101* locus and as a result had 38 protein-encoding genes.

In contrast to the strong conservation of gene content, our analyses of gene order and orientation indicated that synteny is poorly conserved in Peronosporaceae mitogenomes, although several gene blocks are maintained. Our sample of 71 Peronosporaceae mitogenomes contained 28 distinct gene arrangements (fig. 2). Isolates representing the same taxon always shared synteny and, less frequently, synteny was also shared between taxa. Of the 28 gene arrangements recovered, nine were shared between taxa. In most cases, taxa that shared the same gene arrangement belonged to the same numbered clade (e.g., arrangements I and IV). There were three exceptions, two syntenies were shared by members of clades 1 and 4 (i.e., arrangements XX and XXI) with a third shared by members of clades 7, 9, 12, and 15 (i.e., arrangement XXII) (fig. 2).

The extent to which syntenies differ from one another is broadly consistent with the relatedness of the isolates being compared. For isolates representing the same taxon, common interval distances were always the maximum value for the taxon (e.g., 4,284 for *P. agathidicida*, *P. infestans*, and *P. kernoviae*) whereas breakpoint and reversal distances were always minimum values (e.g., both were 0 for *P. agathidicida*, *P. infestans*, and *P. kernoviae*). In contrast, average common interval distances were lower (i.e., 382.34–2,639.23) and average breakpoint (i.e., 3.10–8.99) and reversal (i.e., 1.97–6.64) distances higher for between species comparisons. Moreover, average values for between species comparisons within a numbered clade were more similar to those for isolates representing the same taxon than between clade comparisons (table 2). Similar patterns were also observed at the level of sequence divergence (supplementary fig. S1, Supplementary Material online). Specifically, sequence divergence was moderately correlated with common interval and breakpoint distances (Kendall’s *τ* = 0.205 and 0.251, respectively) whereas the correlation to reversal distances was strong (Kendall’s *τ* = 0.301); in all three cases the relationships were statistically significant (*P* < 0.001).

**Table 2:** Summary of Between Species Comparisons of Genome Structure for the Sampled Peronosporaceae

Clade	Common interval distances			Breakpoint distance			Reversal distance		
	Range	Means		Range	Means		Range	Means	
		Within clade	Between clades		Within clade	Between clades		Within clade	Between clades
Clade 1	378–4,284	3,467.78	2,408.76	0–7	0.89	3.59	0–5	0.44	2.33
Clade 2	416–4,284	3,374.20	2,224.44	0–8	1.03	4.27	0–6	0.53	2.97
Clade 3	416–4,284	4,284.00	2,023.26	0–8	0.00	4.38	0–6	0.00	2.90
Clade 4	378–4,284	3,072.00	2,463.11	0–8	1.75	3.49	0–5	1.13	2.29
Clade 5	378–4,284	4,284.00	1,812.97	0–9	0.00	4.86	0–6	0.00	3.02
Clade 6	270–4,284	3,955.88	1,997.70	0–8	0.00	4.27	0–5	0.00	2.86
Clade 7	214–4,284	2,885.71	1,958.55	0–10	3.02	5.54	0–7	1.55	3.55
Clade 8	378–4,416	4,230.67	2,528.61	0–7	0.89	3.71	0–5	0.44	2.37
Clade 9	378–4,284	4,063.56	2,517.81	0–6	1.33	3.71	0–5	0.89	2.53
Clade 10	382–4,284	4,284.00	1,678.29	0–9	0.00	6.07	0–7	0.00	4.68
Clade 12	378–4,284	4,284.00	2,626.62	0–6	0.00	3.38	0–5	0.00	2.12
Clade 15	378–4,416	2,777.88	2,209.62	0–9	3.18	4.70	0–7	2.29	3.23
Clade 16	458–4,416	4,317.00	1,980.16	2–10	1.00	6.44	1–7	0.50	4.24
*Nothophytophthora*	344–1,394	–	1,281.06	3–12	–	8.99	2–9	–	6.64
*Phytopythium*	214–458	–	382.34	2–6	–	3.10	1–4	–	2.37

### Phylogenetic Analyses

Our final data matrix contained 34 gene partitions from 72 isolates representing 54 members of Peronosporaceae and one of Pythiaceae (supplementary table S2, Supplementary Material online). The matrix was 25,443 aligned nucleotide positions in length, of which 11,225 (44.1%) were variable and 14,218 (55.9%) invariant. Gapped characters composed 0.03% of the matrix (609/1,831,896 characters); 14 of the 34 gene partitions (41.2%) contain gaps, with more than 50% (339/609) of the gaps falling within three partitions (i.e., *rps7*, *rps10*, and *rps11*).

**Fig. 3.**
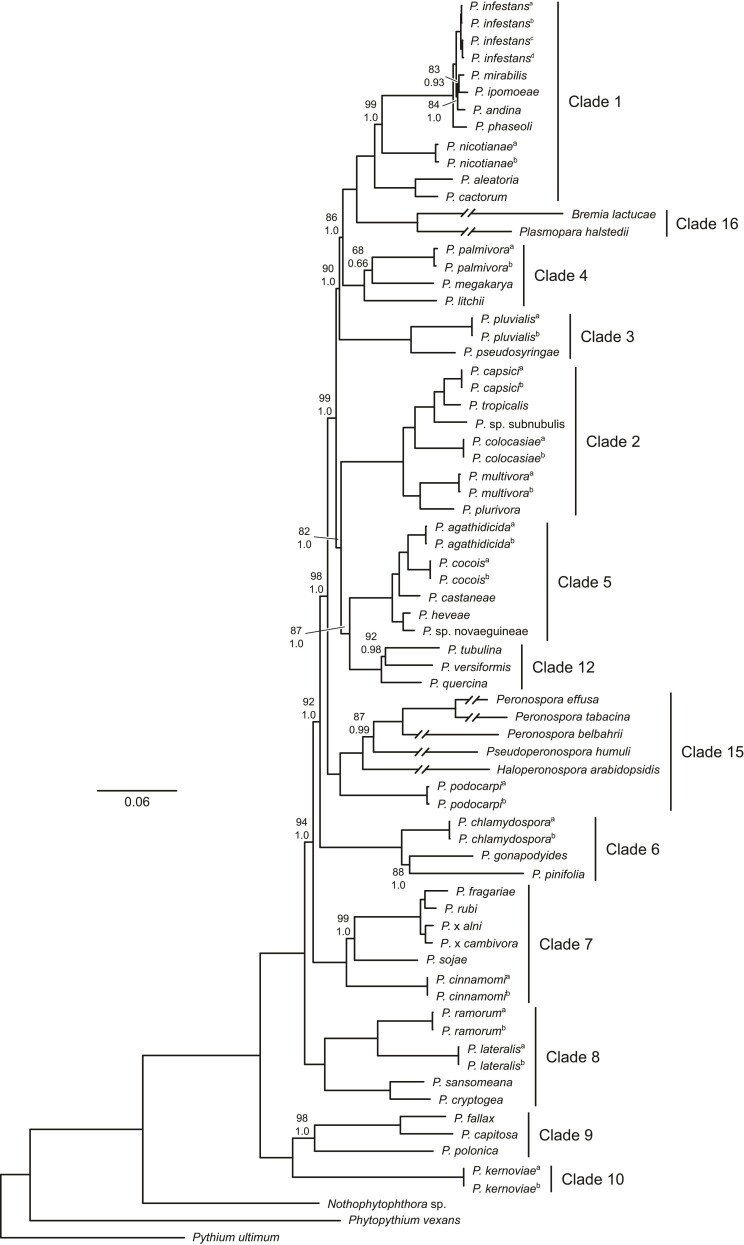
Phylogenetic relationships within Peronosporaceae based on analyses of the combined 34-gene mitochondrial sequence matrix. The topology is that recovered by Bayesian analyses with branch lengths proportional to the mean of the corresponding posterior probability density. Values associated with branches are maximum likelihood bootstrap support (upper) and Bayesian posterior probabilities (lower); values are only reported where bootstrap support was <100%. Superscript letters distinguish isolates of the same species (see supplementary table S1, Supplementary Material online for details) and the major clades recognized by Bourret et al. (2018) are indicated on the right.

Our IQTREE analysis resulted in well-resolved and generally well-supported topology (fig. 3). Specifically, of the 69 internal edges in the topology all but 17 were supported by bootstrap support values of 100% and just one was not strongly supported (i.e., bootstrap support <80% bootstrap). Specifically, the pairing of *P. megakarya* and *P. palmivora* (clade 4) received 68% bootstrap support (fig. 3). All the numbered clades recognized by Bourret et al. (2018) and represented in our sample were very strongly supported as monophyletic (bs > 98%). The relationships amongst the numbered clades were also strongly supported (bs = 82–100%; fig. 3). Relationships within the numbered clades were consistent with those recovered by Bourret et al. (2018) and when multiple isolates of a species were included (e.g., *P. agathidicida*, *P. multivora*) these were consistently recovered and strongly supported (all bs = 100%) as sister.

The sampled pool for each of the replicate Bayesian runs included 60,002 trees with the 95% credible tree set for each including five trees. The topology of the majority-rule consensus for the combined pool of sampled Bayesian trees was the same as that of the maximum likelihood tree. Again this topology was consistent with that of Bourret et al. (2018) with respect to the numbered clades and relationships within them. Our Bayesian analysis also provided very strong support for almost all of the suggested relationships. In this case, all the numbered clades and the relationships amongst them were supported by posterior probabilities of 1.00. Moreover, all but four of the suggested relationships within the numbered clades were also supported by posterior probabilities of 1.00. The exceptions were the *P. andina-ipomoeae*, *P. megakarya*-*palmivora*, *P. tubulina*-*vesiformis*, and *Peronospora*-*Pseudoperonospora* clades for which posterior probabilities were 0.93, 0.66, 0.98, and 0.99, respectively (fig. 3).

Concordance factor values (i.e., gCF and sCF) estimated using IQTREE were typically lower than the bootstrap values for the corresponding branches. The range of values for gCF and sCF were 2.9–100.0 and 33.4–100.0, respectively. In both cases more than half of the values were 50.0 or above; specifically, 59.4% (41/69) for gCF and 58.0% (40/69) for sCF. Indeed, the values of gCF and sCF appear to be strongly correlated (Kendall’s *τ* = 0.765, *P* < 0.001). Both gCF and sCF are also strongly correlated with both branch length and bootstrap values (Kendall’s *τ* = 0.432–0.572, *P* < 0.001) and moderately correlated with posterior probabilities (Kendall’s *τ* = 0.217–0.268, *P* < 0.031) (supplementary fig. S2, Supplementary Material online).

Finally, an evaluation of gene order evolution based on the mitochondrial phylogeny suggested that 48 events were required to explain our sample of Peronosporaceae mitogenomes. These included 38 structural rearrangements (e.g., inversions, transpositions, inverted transpositions), 6 gains and 4 losses (supplementary fig. S3, Supplementary Material online). For structural rearrangements, which were inferred using TreeREx (Bernt et al. 2008), confidence was high for 74.3% (52/70) and low for 11.4% (8/70) of nodes. Consistent with the number and diversity of syntenies we recovered (fig. 2) inferred events were broadly distributed on the phylogeny. Specifically, events were inferred to have occurred on 26.6% (37/139) of the branches with a single event inferred on 78.4% (29/37) branches and between two and four events inferred on the remaining 21.6% (8/37) branches. The 13 numbered clades were each associated with between one (e.g., clades 5 and 12) and seven (e.g., clades 7 and 15) events.

## Discussion

We compiled a data set containing complete mitochondrial genome sequences for 71 members of Peronosporaceae including 44 newly assembled genomes. Our sample increases both the number of recognized clades for which complete mitochondrial genomes are available (i.e., from 9 to 13) and sampling within those clades for which complete mitochondrial genome sequences were already available (e.g., from one to seven genomes for clade 7). This much larger sample provides new insights into the structural diversity of mitogenomes in Peronosporaceae as well as a large data set for phylogenetic analyses.

### Genome Size and Gene Content

Comparisons of the sampled Peronosporaceae mitochondrial genomes suggest that they are similar. For example, with few exceptions both genome size and gene content were comparable across our sample (table 1). However, our analyses indicate a statistically significant difference in mitogenome size between the Peronosporaceae and other oomycete families. Specifically, the Peronosporaceae mitogenomes were smaller (Wilcoxon rank-sum test, *W* = 13, *P* = 0.000012). Despite this overall trend there are exceptions. The *Phytophthora* × *cambivora* mitochondrial genome is comparable in size to those available for Pythiaceae (i.e., 51,184 bp compared with 54,989–59,689 bp) and, at 61,242 bp, the mitochondrial genome of *Ph. vexans* is the largest yet reported for oomycetes.

Mitochondrial gene content is strongly conserved across Peronosporaceae and in almost all cases the mitogenomes are compact with little noncoding sequence (table 1). Typically, the sampled Peronosporaceae mitogenomes contained 39 protein coding and 2 rRNA genes as well as 25 tRNA genes specifying 19 amino acids. However, in seven cases mitochondrial gene content has been expanded by between 1 and 46 genes (fig. 2). For example, the *P. kernoviae* mitogenome contained two additional tRNA genes (i.e., arrangement XXVI) and for *P. pinifolia* there were six additional genes, four protein encoding and two tRNA genes (i.e., arrangement XIV). The largest increase was for *Ph. vexans* (i.e., arrangement XXVIII) where there were 27 additional protein encoding, 2 additional rRNA, and 17 additional tRNA genes. Increased protein-encoding gene content was associated with larger genome sizes. Specifically, all four Peronosporaceae mitogenomes that contained more than 39 protein-coding genes were more than 43,000 bp in length (i.e., *P. alni*, *P. pinifolia*, *P*. *rubi*, and *Ph. vexans*). In some cases, however, larger genome size was not associated with expanded gene content. For example, the *P.* × *cambivora* mitochondrial genome had the typical 39 protein coding, 2 rRNA, and 25 tRNA genes but was more than 5,000 bp larger than all but one (i.e., *Ph. vexans*) of the mitogenomes in our data set. The repeated DNA sequences responsible for the larger size of the genome were entirely noncoding.

Our analyses included 15 taxa for which mitogenome sequences from two or more isolates were included. For three of these taxa, both sequences were the same length, while for the remaining 12 taxa the sequences differed in length by between 2 and 2,058 bp. The majority of the length differences were explained by multiple indels, just three cases involved single indels (e.g., a 180 bp indel in *P. ramorum*). In addition to different numbers of indels (i.e., one to many), their size (i.e., a single nucleotide to >50 nucleotides [nt]), distribution (i.e., distributed throughout the genome or clustered), and underlying sequence (i.e., unique or repeated sequences) also varied. These observations suggest that a combination of molecular evolutionary processes contributes to mitogenome size variation within taxa.

### Genome Structure

Previous studies have suggested that although inverted repeats are an important feature of both Pythiales and Saprolegniales mitogenomes they are not typical of *Phytophthora* mitogenomes. For example, of the nine *Phytophthora* mitochondrial genomes compared by Yuan et al. (2017) only that of *P. ramorum* contained inverted repeats. However, in our data set inverted repeats longer than 150 bp were a feature of approximately one-quarter of the taxa, including 11 members of *Phytophthora.* Smaller inverted repeats (i.e., *Hyaloperonospora*) and direct repeats (e.g., *B. lactucae*, *Pe. tabacina*) were also identified.

In our analyses, the distribution of inverted repeats was nonrandom with respect to genome size. Mitogenomes shorter than 38,500 bp in length did not contain inverted repeats, whereas more than half (56.8%) of those longer than 38,500 bp and all but one of those longer than 43,000 bp contained them. The distribution of inverted repeats also appears to be nonrandom with respect to the phylogeny. Specifically, with a couple of exceptions, clades in which inverted repeats were absent (i.e., clades 1–5, 12 and *Nothophytophthora*) formed a monophyletic group. The exceptions were clade 16, which despite containing species that possessed mitogenomes with inverted repeats was placed within the group lacking them and *Nothophytophthora* that falls outside this same group. Further work is needed but given the similarity of the inverted repeats in *Phytopythium* and *Pythium*, it is conceivable that the mitogenome of the earliest Peronosporaceae possessed large inverted repeats. Despite this, inverted repeats were uncommon amongst the Peronosporaceae mitogenomes examined and, even when present, were much smaller than those of *Phytopythium* or *Pythium* (i.e., >5,442 bp vs. 21,950–24,191). We suggest that large inverted repeats were lost early in the evolution of Peronosporaceae and that smaller repeats have subsequently arisen in multiple clades. An alternative explanation is that smaller repeats arose repeatedly via progressive reduction of the larger ancestral repeats. However, in at least some cases (i.e., arrangements XIV and XV) the smaller inverted repeats contained genes not found in the larger repeats of *Phytopythium* or *Pythium*, making this explanation less likely.

Our analyses suggest structural rearrangements have played an important role in the evolution of Peronosporaceae mitochondrial genomes. Specifically, based on gene order comparisons we identified 28 structurally distinct mitogenomes among the 55 taxa we sampled. Each of the 15 named or numbered clades that we sampled was represented by at least one, and typically two or more, unique arrangements. For example, all five sampled clade 5 taxa shared the same gene order whereas each of the six sampled clade 7 species had different gene orders. Most commonly gene orders were restricted to specific named or numbered clades (e.g., arrangements I–V). However, we identified three arrangements shared by two or more of the numbered clades (i.e., arrangements XX–XXII). For example, arrangement XXII was shared by *P. sojae* (clade 7), *P. polonica* (clade 9), all members of clade 12, and 2 of 3 *Peronospora* species (clade 15).

Pairwise comparisons of the 28 structurally distinct syntenies suggest that the degree of difference between syntenies varies. Some mitogenomes differed by a single change. For example, a single inversion (e.g., arrangements V and VII differ by an inversion of *nad5*–*nad6*–*tRNA-Arg*), loss (e.g., loss of *ymf101* in I relative to XX), or gain (e.g., gain of a second *nad4L* copy in XIX relative to XVIII). In others, several changes are needed to explain the differences. For example, XVIII and XXII appear to differ on the basis of two overlapping inversions; a large one involving a segment containing 25 genes (i.e., *tRNA-Arg* to *cob*) and a smaller one involving a 10 gene segment (i.e., *rps7* to *nad7*). The most distinctive mitogenomes were arrangements XI, XXVI, XXVII, and XXVIII. In most Peronosporaceae the two mitochondrial rRNA genes group together with *rpl5*, *rpl14*, and eight tRNA genes. However, in these four mitogenome arrangements the two rRNA genes were separated by 28–37 protein coding and tRNA genes. In *Nothophytophthora* sp. (XXVII) and *Ph. vexans* (XXVIII), the two *rpl* genes fell next to the ssRNA gene whereas in *P. kernoviae* (XXVI) and *P. podocarpi* (XI), they are well separated (fig. 2). The distinctiveness of *P. kernoviae*, *P. podocarpi*, and *Nothophytophthora* sp. is consistent with comparatively low average common interval distances and comparatively high average breakpoint and reversal distances for these three species. In contrast, although average common interval distances were very low for *Ph. vexans* (i.e., 214–458 cf., 1,096–4,416), average breakpoint and reversal distances were more similar to those of the other species (i.e., 2–6 cf., 0–12 and 1–4 cf., 0–9, respectively). This likely reflects the presence of large inverted repeat in *Ph. vexans*.

Further sampling is required to fully characterize the structural diversity of Peronosporaceae mitogenomes. However, our analyses suggest several insights. First, different evolutionary processes appear to be acting on mitogenome structure within and between taxa. Specifically, within taxa we only observed nucleotide substitutions and length differences whereas between taxa structural rearrangements were also observed. Second, the rearrangements that underpin differences in mitogenome structure between taxa are not uniformly distributed across the genome. Instead, the Peronosporaceae mitogenome appears to be composed of two distinct segments. One, containing 30 protein encoding and ribosomal genes, was stable with respect to gene content and order. The other varied markedly in both gene content and order. Containing 35–81 genes all of the structural rearrangements that differentiated the 28 observed syntenies were located within this segment. Despite the variability within this block, all the examined mitogenomes shared several features. For example, typically the tRNA genes have maintained their positions relative to their immediate neighbors (e.g., all the mitogenomes contained the gene blocks *rps10*–*tRNA-Arg*–*tRNA-Gln–tRNA-Ile*–*tRNA-Val–rps12* and *atp6*–*tRNA-Asp*–*nad3*). This implies rearrangements have more commonly occurred between protein encoding than ribosomal genes.

Finally, our results are not consistent with the suggestion that inverted repeats stabilize organellar genomes against intramolecular homologous recombination (e.g., Adelberg and Bergquist 1972; Hudspeth et al. 1983). Specifically, we recovered between two and five syntenies from clades containing mitogenome sequences with inverted repeats but three or fewer arrangements from those in which mitogenomes did not contain inverted repeats (clade 10 and *Phytopythium* for which we had only one representative were not considered). Further work is needed but there are several possible explanations. One possibility is that inverted repeats act as rearrangement “hotspots” (e.g., Martin et al. 2007). In this case, mitogenomes containing inverted repeats may retain the potential for structural change. Alternatively, genome size may determine whether rearrangements are likely. For example, loss of the inverted repeats could lead to genomes that are so compact that rearrangements become unlikely. Time may also play a role. That is, clades with smaller numbers of genome arrangements may have been diversifying for a shorter period of time than those with larger numbers.

### Phylogenetic Analyses

The phylogenetic relationships suggested by our analyses of combined mitochondrial protein-coding gene sequences were broadly consistent with those reported previously (e.g., Blair et al. 2008; Yang et al. 2017; Bourret et al. 2018). Specifically, we sampled 13 of the 16 numbered clades recognized by Bourret et al. (2018) and in our phylogenetic analyses, each was recovered with very strong support (i.e., posterior probabilities = 1.0, bootstrap values >98%). Relationships within the numbered clades were also consistent with those reported by Bourret et al. (2018). Moreover, all but one of these relationships, the pairing of *P. megakarya*-*palmivora* (posterior probability = 0.66, bootstrap value = 68%) received moderate to very strong support in both analyses. The placements of *Nothophytophthora* sp. and *Ph. vexans* were also consistent with previous analyses (e.g., Jung et al. 2017b). Specifically, *Phytopythium* branched first with *Nothophytophthora* sister to the clade containing *Phytophthora* and the downy mildews.

Although previous analyses have provided strong support for the named and numbered clades, relationships among them have not been consistently resolved or well supported. For example, Martin et al. (2014) conducted analyses of mitochondrial, nuclear, and concatenated matrices finding four different resolutions of relationships for clade 2. At least, two of these had been recovered in previous analyses (e.g., Blair et al. 2008; Yang et al. 2017). Bourret et al. (2018) highlighted hybridization, incomplete lineage sorting and analytical errors as potential explanations for discordance between mitochondrial and nuclear topologies. Our analyses suggest an additional insight. Despite strong support for relationships amongst the named and numbered clades (i.e., posterior probabilities = 1.0, bootstrap values = 82–100%), concordance factor values for all but two of these relationships (i.e., those involving *Phytopythium* and *Nothophytophthora*) fell below 50%. Short branches can contribute to low concordance factor values and we found strong correlations between each of the concordance factors and branch length. More specifically, 45.5% (10/22) of branches representing relationships between the numbered clades were also amongst the shortest 50% of internal branches in our topology and for 80.0% (8/10) of these, the value of one or both concordance factors fell below 50%. Although further analyses are needed this observation implies that the numbered clades arose over a relatively short period early in the evolution of Peronosporaceae. If so, then even in the absence of hybridization or incomplete lineage sorting these relationships are likely to be difficult to reconstruct, especially with relatively limited data (Hendy and Penny 1989).

An analysis of genome rearrangements based on our topology is consistent with a complex history of structural evolution for Peronosporaceae mitogenomes. Our analysis suggested 48 events with up to four on a single branch (i.e., that leading to *Nothophytophthora* sp.) and seven within a single clade (i.e., clades 7 and 15). However, our taxon sampling is limited and we remain cautious about inferring ancestral gene order. Although further sampling is needed similarities between the mitogenomes of *Nothophytophthora*, *Phytopythium*, and *Py. ultimum* (Pythiaceae) may provide some insights. Specifically, all three of these taxa share a gene order in which the ssRNA, *rpl5*, r*pl14*, and several tRNA genes are well separated from the lsRNA gene. Additionally, *Phytopythium* has a large inverted repeat similar, although not identical in gene content, to that of *Py. ultimum*. In both cases, none of the other sampled Peronosporaceae have equivalent features (e.g., inverted repeats of other Peronosporaceae were much smaller and contained fewer genes). A working hypothesis is that the mitogenomes of the earliest Peronosporaceae possessed these two features.

Our phylogenetic analyses combined broad samples of taxa and mitochondrial protein-coding sequences. Although the resulting phylogeny provides evolutionary insights it is unlikely to fully describe the underlying species tree. However, a robust mitochondrial phylogeny could provide a framework for further analyses. One possible use would be in formal tests of hybridization (e.g., Joly et al. 2009; Joly 2012). There are numerous examples of both natural (e.g., Bonants et al. 2000; Nirenberg et al. 2009; Goss et al. 2011) and synthetic (e.g., Goodwin and Fry 1994; Donahoo et al. 2008) *Phytophthora* hybrids. Moreover, in this genus hybridization is associated with host range shifts (e.g., Ersek et al. 1995; Man in ‘t Veld et al. 2006) and a role for hybridization in species diversification has been hypothesized (Bertier et al. 2013). Testing for hybridization involves gene tree incongruence, which could be evaluated using phylogenies based on mitochondrial genome sequences. Mitogenome sequences might also be used to estimate the timeframe over which Peronosporaceae have evolved. Matari and Blair (2013) used a multilocus nuclear data set to estimate divergence times for the oomycetes and complete mitogenome sequences have been used for age estimation in *Phytophthora* (e.g., Martin et al. 2016; Winkworth et al. 2021). Matrices of mitogenome sequences are relatively long and this is encouraging in terms of resolving the timeframe over which the Peronosporaceae have evolved.

## Materials and Methods

### Fungal Culture and DNA Extraction

We obtained single isolates of *Nothophytophthora* sp., *P. captiosa*, *P. chlamydospora*, and *P. fallax* as well as two of *P. palmivora* from the International Collection of Microorganisms from Plants (ICMP) culture collection (supplementary table S1, Supplementary Material online). Isolates were cultured on *Phytophthora-*selective media (Jeffers and Martin 1986) in the dark at 18**°**C for up to 10 days. Agar plugs were excised from each plate and incubated overnight at 56°C with 180 µl ATL buffer (Qiagen, Hilden, Germany) and 20 µl proteinase K (20 mg/ml; Qiagen). The tubes were then centrifuged with genomic DNA extracted from the supernatant using the QIAcube® instrument and the QIAamp® DNA mini QIAcube kit (Qiagen).

### Library Preparation and Genome Sequencing

Shotgun sequencing libraries were prepared from each of the DNA extracts by the Massey Genome Service (Palmerston North, New Zealand) using Illumina Nextera DNA library preparation kits (Illumina, Inc., San Diego, CA, USA). The Massey Genome Service also performed 2 × 250 base pair (bp), paired-end DNA sequencing on Illumina MiSeq instruments and quality assessed reads using a combination of SolexaQA (Cox et al. 2010) and FastQC (Andrews 2010).

### Mitochondrial Genome Assembly and Annotation

We assembled complete mitochondrial genome sequences for 44 isolates representing 34 members of the Peronosporaceae. In addition to the six newly sequenced isolates, we assembled 38 genomes using data available from the NCBI Sequence Read Archive (supplementary table S1, Supplementary Material online).

For all 44 isolates, we conducted de novo assemblies of quality-assessed sequence reads using idba_ud (Peng et al. 2012). The resulting contigs were then filtered against a library of publicly available Peronosporaceae mitochondrial genome sequences (supplementary table S1, Supplementary Material online) using BLAST (Altschul et al. 1997). Draft genomes were assembled from this mitochondrial subset using the assembly tools implemented in Geneious R9 (Kearse et al. 2012). To evaluate drafts, we used BWA (Li and Durbin 2009) to map the original quality-assessed sequence reads to the corresponding draft genome. Inconsistencies in read coverage were interpreted as assembly errors and draft sequences revised as appropriate.

The final mitochondrial genome sequences were annotated on the basis of sequence similarity using a combination of Geneious and DOGMA (Wyman et al. 2004). For each genome we also identified repeated sequences using the Repeat Finder tool in Geneious; for these searches we considered only perfect repeats with a minimum repeat length of 150 nt.

### Data Matrices and Phylogenetic Analyses

For phylogenetic analyses, we assembled multiple sequence alignments for the 39 common protein-coding loci. Initial alignments were generated using ClustalO (Sievers et al. 2011) and edited in Mesquite v3 (Maddison and Maddison 2019) to remove portions where <50% of the sequences were represented or where alignments were otherwise ambiguous. For phylogenetic analyses, a concatenated matrix containing 34 loci was compiled. Due to high levels of length and sequence variation we excluded alignments for the four hypothetical proteins (i.e., *ymf98*, *ymf99*, *ymf100*, and *ymf100*) and the sec-independent transporter protein from further analyses.

Prior to maximum likelihood and Bayesian analyses, we first used the Bayesian information criterion (BIC; Schwarz 1978) as implemented in jModelTest 2.2 (Guindon and Gascuel 2003; Posada 2008) to identify best-fit substitution models for each gene partition. We then performed a partitioned maximum likelihood search using IQ-TREE v2.1.3 (Minh et al. 2020a; Chernomor et al. 2016). For this search, we applied best-fit substitution models and support for relationships was evaluated using 1,000 bootstrap replicates. Bayesian searches were performed using MrBayes 3.2 (Ronquist and Huelsenbeck 2003). In this case, we conducted two identical searches, each 2.5 × 10^7^ generations in length and sampled every 1.0 × 10^3^ generations. For each run, we determined burn-in using convergence diagnostics and samples drawn prior to stationarity discarded. Posterior distributions of parameters were examined using Tracer v1.7 (Rambaut et al. 2018) and consensus trees visualized with FigTree v1.4.

To examine conflict within our data set, we calculated a pair of concordance factors as implemented in IQ-TREE (Minh et al. 2020b). For any given branch in a reference topology (e.g., from an analysis of concatenated sequences in a phylogenomic study), the gene concordance factor (gCF) is the percentage of gene trees (e.g., from analyses of the individual sequence partitions) that contain the same branch and the site concordance factor (sCF) is the percentage of informative alignment positions across the concatenated matrix that support the branch. Our analysis made use of the topology from the partitioned maximum likelihood search as well as those from similar searches for each of the 34 mitochondrial gene partitions. We used Kendall’s *τ*, as implemented in R (R Core Team 2021), to evaluate whether the two concordance factors were correlated as well as whether they were correlated with the length of and support values for the corresponding branches.

### Comparisons of Genome Structure

We evaluated the overall structure of the Peronosporaceae mitochondrial genomes in terms of gene order, gene orientation and repeats. To facilitate these analyses, we first standardized the annotations for all 71 included mitogenome sequences. We considered a core set of 39 protein coding, 25 tRNA, and 2 rRNA genes common to the majority of the Peronosporaceae mitogenome sequences. In most cases, departures from this core set involved duplications of one or more genes or inclusion of open reading frames. Duplicated core genes and those open reading frames with high similarity to one of the core genes (e.g., the *P. infestans orf217* was similar to *ymf99*) were retained. We excluded gene annotations not amongst the core set (e.g., *ymf96*) and open reading frames not similar to one of the core genes. We also reoriented all the genomes so that the large ribosomal RNA subunit was the forward orientation and that the first residue of the genome corresponded to the first residue of this gene.

To compare the overall structural similarity of Peronosporaceae mitochondrial genomes we examined gene order and orientation using the common interval approach (Uno and Yagiura 2000; Heber and Stoye 2001) as implemented in CREx (Bernt et al. 2007). CREx does not allow for duplicated genes and we used two approaches to address multiple gene copies. For genes that existed as multiple copies in all the sampled genomes (e.g., *tRNA-Arg* and *tRNA-Ser*) we labeled each copy with a unique identifier, in all these cases we were able to distinguish copies based on their relative position in the genome. For genes duplicated as part of a repeat in one or a few mitogenomes (e.g., *atp6*, *tRNA*-*Asp*, *nad3*, *nad5*, *nad6*, and *tRNA*-*Arg* in *P*. *pinifolia*) we removed one of the repeats prior to analysis. In each case, we retained the copy of the repeat that minimized the number of required changes. For this analysis, we used default parameters and compared all genome pairs based on common interval, breakpoint and reversal distances. We used Kendall’s *τ*, as implemented in R (R Core Team 2021), to evaluate whether common interval, breakpoint, and reversal distances between pairs of accessions were correlated with sequence divergence (i.e., *p* distances).

We also performed an ancestral character state reconstruction of gene order using TreeREx (Bernt et al. 2008). In this case, we used our maximum likelihood tree topology, the same gene orders as for CREx and default parameters.

## Supplementary Material

Supplementary data are available at *Genome Biology and Evolutio*n online.

## Supplementary Material

evac049_Supplementary_Data

## Data Availability

The data underlying this article are available in the GenBank Nucleotide Database at https://www.ncbi.nlm.nih.gov/nucleotide, and can be accessed using the accession numbers listed in Supplementary material, Supplementary Material online.
