# Supplementary material for: Comparative Analyses of Complete Peronosporaceae (Oomycota) Mitogenome Sequences—Insights into Structural Evolution and Phylogeny
Source: Genome Biol Evol. 2022 Apr 14;14(4):evac049. doi: 10.1093/gbe/evac049 (PMC9020773; doi:10.1093/gbe/evac049)
Supplement: evac049_Supplementary_Data [file evac049_supplementary_data.zip › Winkworth_etal_SupplementaryFigures.pdf]

Supplementary Figure Legends

Supplementary Fig 1. Scatterplots describing the relationship between sequence divergence and differences in gene order for pairs of Peronosporaceae mitogenomes. Three measures of gene order difference were evaluated. Panel A. Common interval distance; Panel B. Reversal distance; Panel C. Breakpoint distance. Regresion lines are black and associated confidence intervals shaded.

Supplementary Fig 2. Scatterplots describing relationship between concordance factors and inferred branch lengths for our Peronosporaceae mitochondrial protein coding gene data matrix. Panels A and B, gene concordance factors; Panels C and D, site concordance factors. Point colour reflects statistical support for the corresponding branch in the phylogeny; bootstrap values (Panels A and C) and posterior probabilities (Panels B and D).

Supplementary Fig 3. Ancestral pattern of gene order evolution in Peronosporaceae as inferred by TreeREx. Reconstructions at nodes marked in green were consistent, those marked in yellow were k-consistent and those in red were inconsistent. Rearrangement types are denoted as empty rectangle, transposition; shaded rectangle, inversion and transposition; shaded star, inversion; shaded hexagon, tandem-duplication-random-loss (TDRL). Empty or shaded triangles indicate inferred losses or gains, respectively. These were added manually using a parsimony criterion. Superscript letters distinguish isolates

of the same species (see supplementary table 1 for details) and the major clades recognised by Bourret *et al.* (2018) are indicated on the right.

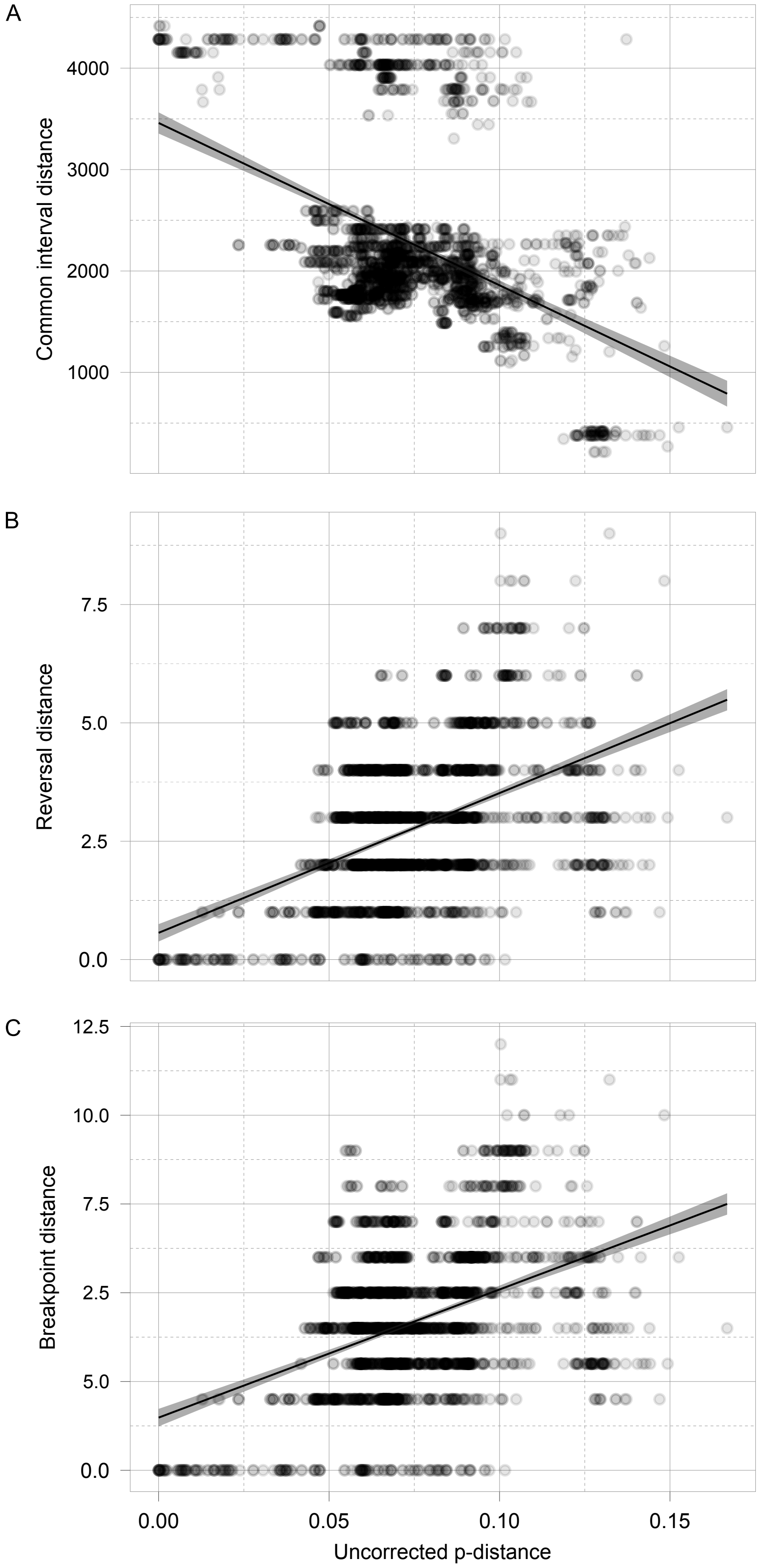
A
4000
3000
2000
1000
Common interval distance
B
7.5
5.0
2.5
0.0
Reversal distance
C
12.5
10.0
7.5
2.5
5.0
0.0
Breakpoint distance
0.00
0.05
0.10
0.15
Uncorrected p-distance

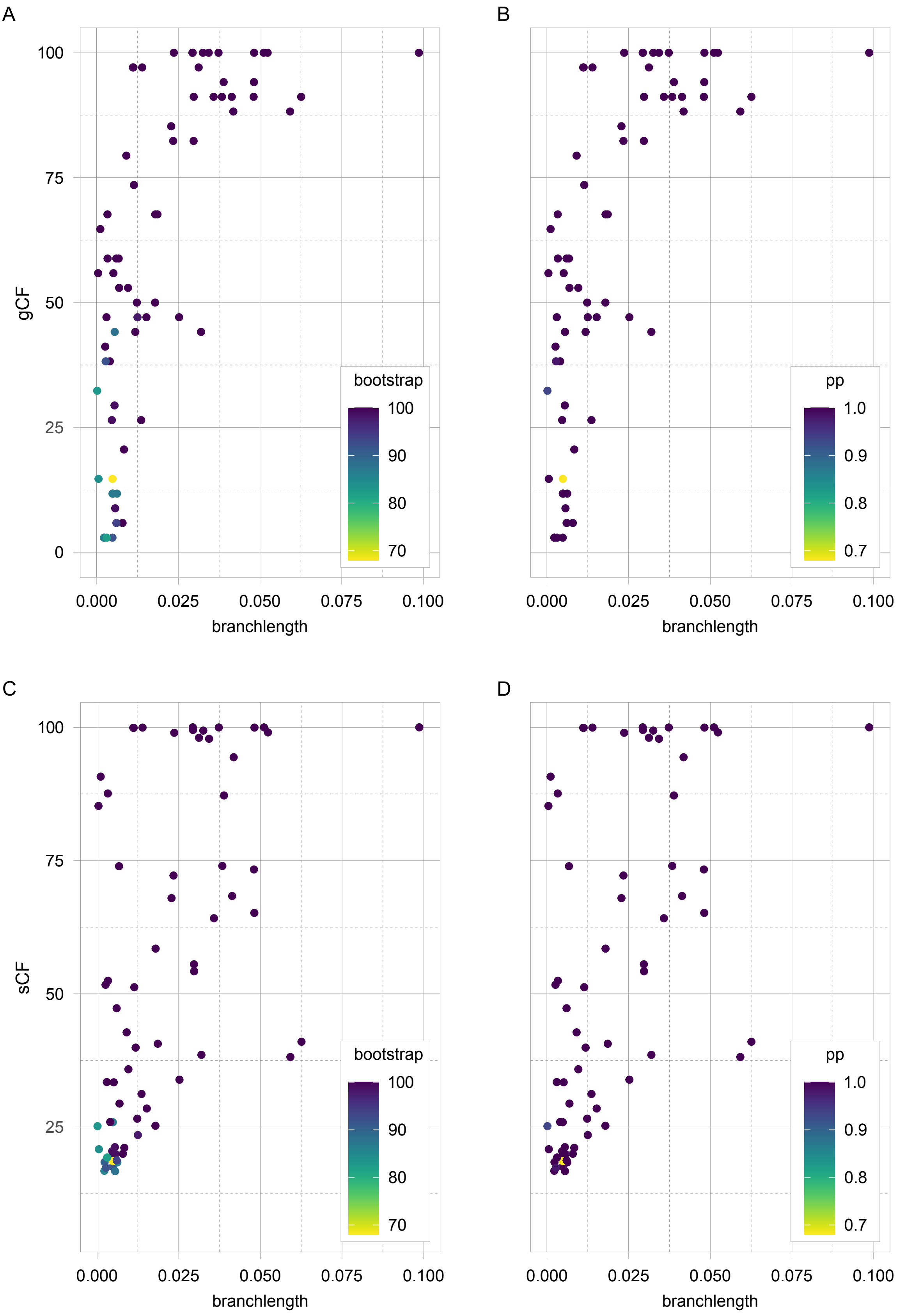
A
B
C
D
gCF
sCF
branchlength
bootstrap
pp
100
90
80
70
1.0
0.9
0.8
0.7
0.000
0.025
0.050
0.075
0.100
0
25
50
75

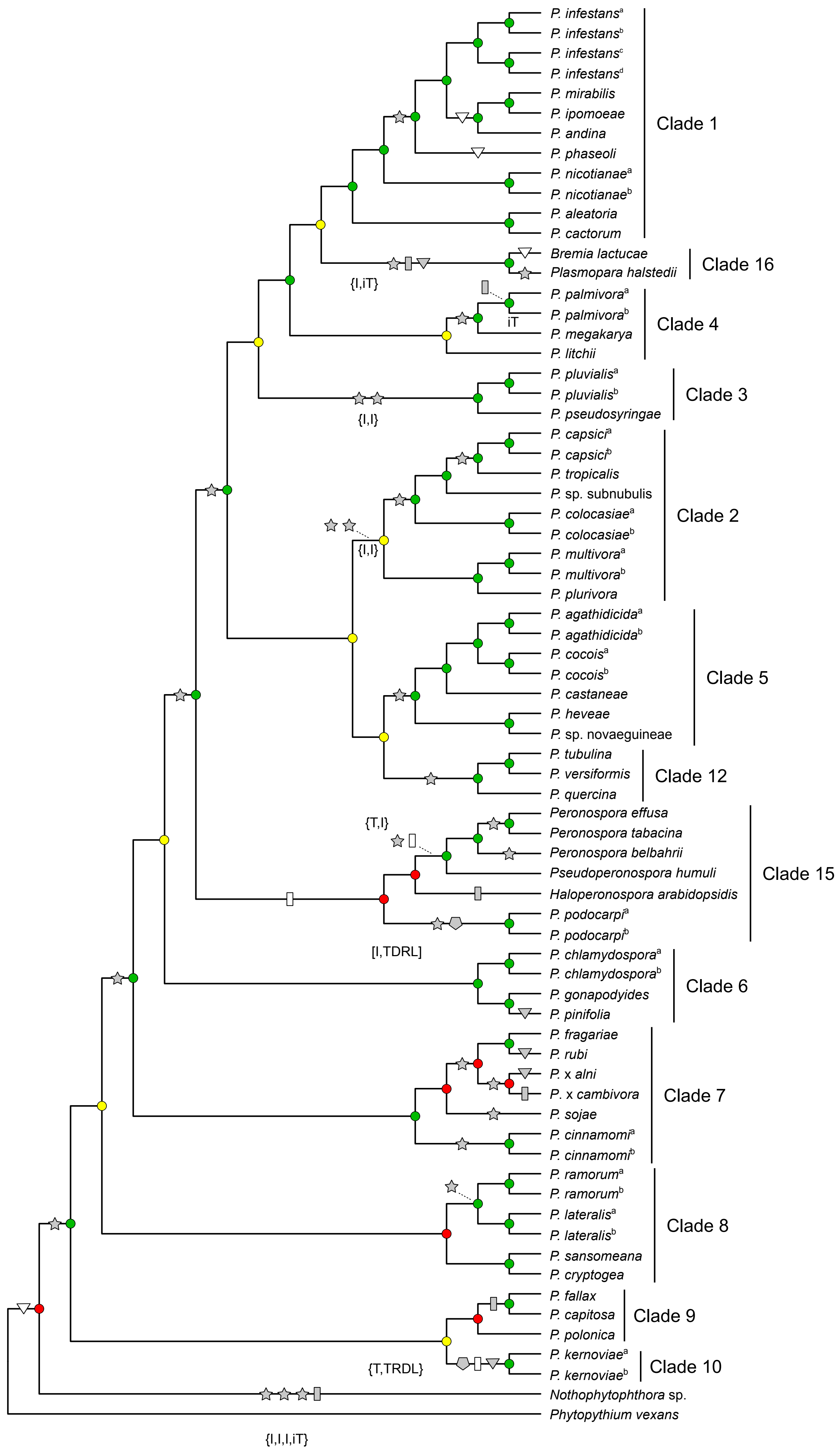

P. infestans[a]
P. infestans[b]
P. infestans[c]
P. infestans[d]
P. mirabilis
P. ipomoeae
P. andina
P. phaseoli
P. nicotianae[a]
P. nicotianae[b]
P. aleatoria
P. cactorum
Clade 1
Bremia lactucae
Plasmopara halstedii
Clade 16
{I,iT}
P. palmivora[a]
P. palmivora[b]
iT
P. megakarya
P. litchii
Clade 4
P. pluvialis[a]
P. pluvialis[b]
P. pseudosyringae
Clade 3
{I,I}
P. capsici[a]
P. capsici[b]
P. tropicalis
P. sp. subnubulis
P. colocasiae[a]
P. colocasiae[b]
{I,I}
P. multivora[a]
P. multivora[b]
P. plurivora
Clade 2
P. agathidicida[a]
P. agathidicida[b]
P. cocois[a]
P. cocois[b]
P. castaneae
P. heveae
P. sp. novaeguineae
Clade 5
P. tubulina
P. versiformis
P. quercina
Clade 12
{T,I}
Peronospora effusa
Peronospora tabacina
Peronospora belbahrii
Pseudoperonospora humuli
Haloperonospora arabidopsidis
P. podocarpi[a]
P. podocarpi[b]
Clade 15
[I,TDRL]
P. chlamydospora[a]
P. chlamydospora[b]
P. gonapodyides
P. pinifolia
Clade 6
P. fragariae
P. rubi
P. x alni
P. x cambivora
P. sojae
P. cinnamomi[a]
P. cinnamomi[b]
Clade 7
P. ramorum[a]
P. ramorum[b]
P. lateralis[a]
P. lateralis[b]
P. sansomeana
P. cryptogea
Clade 8
P. fallax
P. capitosa
P. polonica
Clade 9
P. kernoviae[a]
P. kernoviae[b]
Clade 10
{T,TRDL}
Nothophytophthora sp.
Phytopythium vexans
{I,I,I,iT}
