# Supplementary material for: Comparative Analyses of Complete Peronosporaceae (Oomycota) Mitogenome Sequences—Insights into Structural Evolution and Phylogeny
Source: Genome Biol Evol. 2022 Apr 14;14(4):evac049. doi: 10.1093/gbe/evac049 (PMC9020773; doi:10.1093/gbe/evac049)
Supplement: evac049_Supplementary_Data [file evac049_supplementary_data.zip › Winkworth_etal_SupplementaryTablesrev.pdf]

Supplementary Table 1. Taxa, authorities, isolate, GenBank accession numbers and references for accessions included in this study.

Species and authority	Isolate	Genbank Accession	Reference
Bremia lactucae Regel	–	NC_040179	Martin *et al.* (2018)[e]
Hyaloperonospora arabidopsidis (Gäum.) Göker, Riethm., Voglmayr, Weifl & Oberw.	Noks1	BK011976	This study
Nothophytophthora sp. T. Jung, Scanu, Bakonyi & M. Horta Jung	ICMP 18719	MW648547	This study
Peronospora belbahrii Thines	HI01	BK011978	This study
Peronospora effusa (Grev.) Rabenh.	–	MH142315	Fletcher *et al.* (2018)
Peronospora tabacina Adam	–	KT893455	Derevnina *et al.* (2015)
Phytophthora × *alni* Brasier & S.A.Kirk	CBS 117376	BK059194	This study
Phytophthora × *cambivora* (Petri) Buisman	CBS 114087	BK059195	This study
Phytophthora agathidicida[a] B.S. Weir, Beever, Pennycook & Bellgard	ICMP 16471	MN883601	Winkworth *et al.* (2021)
P. agathidicida[b]	ICMP 18244	MT032126	Winkworth *et al.* (2021)
Phytophthora aleatoria P.M.Scott, R.McDougal & P.M.Taylor	NZFS 4037	BK059193	This study
Phytophthora andina Adler & Flier	–	NC_015619	Lassiter *et al.* (2015)
Phytophthora cactorum (Lebert & Cohn) J. Schröt.	10300	BK011979	This study
Phytophthora captiosa M.A. Dick & Dobbie	ICMP 17567	MN883606	This study
Phytophthora capsici[a] Leonian	Taiwanpc33e	BK012087	This study
P. capsici[b]	Taiwanpc377	BK012088	This study
Phytophthora castaneae Katsura & K. Uchida	ICMP 19450	MN883602	Winkworth *et al.* (2021)
Phytophthora chlamydospora[a] Brasier & Hansen	ICMP 16726	MN883607	This study
P. chlamydospora[b]	–	CM022726	McGowan *et al.* (2020)
Phytophthora cinnamomi[a] Rands	NZFS 3750	BK011982	This study
P. cinnamomi[b]	CBS 144.22	BK012089	This study
Phytophthora cocois[a] B.S. Weir, Beever, Pennycook, Bellgard & J.Y. Uchida	ICMP 16949	MN883603	Winkworth *et al.* (2021)
P. cocois[b]	ICMP 19685	MT032127	Winkworth *et al.* (2021)
Phytophthora colocasiae[a] Racib.	SB9	BK011983	This study
P. colocasiae[b]	BC13	BK012090	This study
Phytophthora cryptogea Pethybr. & Laff.	CBS 418.71	BK011984	This study
Phytophthora fallax Dobbie & M. A. Dick	ICMP 17563	MN883608	This study
Phytophthora fragariae Hickman	CBS 209.46	BK011985	This study
Phytophthora gonapodyides (H. E. Petersen) Buisman	–	CM022728	McGowan *et al.* (2020)
Phytophthora heveae A.W. Thomps.	ICMP 19451	MN883604	Winkworth *et al.* (2021)
Phytophthora infestans[a] (Mont.) de Bary	–	AY894835	Avila-Adame *et al.* (2006)
P. infestans[b]	–	NC_002387	Lang & Forget (1992)
P. infestans[c]	–	AY898627	Avila-Adame *et al.* (2006)
P. infestans[d]	–	AY898628	Avila-Adame *et al.* (2006)
Phytophthora ipomoeae Flier & Grünwald	–	NC_015622	Lassiter *et al.* (2015)
Phytophthora kernoviae[a] Brasier	00844/4	BK011986	This study

P. kernoviae[b]	00629/1	BK012091	This study
Phytophthora lateralis[a] Tucker & Milbrath	CBS 168.42	BK011987	This study
P. lateralis[b]	SMST21	BK012092	This study
Phytophthora litchii (C.C. Chen ex W.H. Ko, H.S. Chang, H.J. Su, C.C. Chen & L.S. Leu) Voglmayr, Göker, Riethm. & Oberw.	SHS3	BK011980	This study
Phytophthora megakarya Brasier & Griffin	Pm2	BK059191	This study
Phytophthora mirabilis Galindo & H.R. Hohl	–	NC_015606	Lassiter *et al.* (2015)
Phytophthora multivora[a] P.M. Scott & T. Jung	NZFS 3448	BK011988	This study
P. multivora[b]	NZFS 3378	BK012093	This study
P. nicotianae[a] Breda de Haan	P1976	BK011990	This study
P. nicotianae[b]	CJ01A1	BK012094	This study
Phytophthora palmivora[a] (E.J. Butler) E.J. Butler	ICMP 17709	MN883609	This study
P. palmivora[b]	ICMP 14517	MT032128	This study
P. phaseoli Thaxter	–	NC_015616	Lassiter *et al.* (2015)
Phytophthora pinifolia Alv. Dur·n, Gryzenh. & M.J. Wingf.	CBS 122922	BK011991	This study
Phytophthora plurivora Jung & Burgess	AV1007	BK059192	This study
Phytophthora pluvialis[a] Reeser, Sutton & Hansen	LC9-1	BK011992	This study
P. pluvialis[b]	NZFS 3000	BK012095	This study
Phytophthora podocarpi[a] sp. nov.	NZFS 3642	BK011993	This study
P. podocarpi[b]	NZFS 3727	BK012097	This study
Phytophthora polonica Belbahri et al.	–	NC_029397	Jastrzebski *et al.* (2015)[e]
Phytophthora pseudosyringae T. Jung and T.I. Burgess	–	CM022727	McGowan *et al.* (2020)
Phytophthora quercina T. Jung and T.I. Burgess	ST_20190808	BK014412	This study
Phytophthora ramorum[a] Werres, De Cock & Man in 't Veld	–	EU427470	Martin (2008)
P. ramorum[b]	–	NC_009384	Martin *et al.* (2007)
Phytophthora rubi (W.F. Wilcox & J.M. Duncan) W.A. Man in 't Veld	pd010150015038	BK014413	This study
Phytophthora sansomeana E.M. Hansen & Reeser	–	NC_045089	Cai & Scofield (2020)
Phytophthora sojae Kaufm. & Gerd.	–	NC_009385	Martin *et al.* (2007)
Phytophthora sp. novaeguineae	ICMP 19637	MN883605	Winkworth *et al.* (2021)
Phytophthora sp. subnubulis	LT5395	BK012096	This study
Phytophthora tropicalis Aragaki & J. Y. Uchida	LT5761	BK011981	This study
Phytophthora tubulina Jung, Cech, Horta Jung & Bakonyi	ST_20191127	BK014414	This study
Phytophthora versiformis Paap and Burgess	ST_20190930	BK014415	This study
Phytopythium vexans (de Bary) Abad, De Cock, Bala, Robideau, Lodhi & Le vesque	CBS 119.80	BK059196	This study
Plasmopara halstedii (Farl.) Berl. & De Toni	710	BK012098	This study

Pseudoperonospora humuli (Miyabe & Takah.) G.W. Wilson	–	NC_042478	Rahman *et al*. (2019)
Pythium ultimum Trow	–	NC_014280	Levesque *et al*. (2010)

[a-d] Superscript letters distinguish isolates of the same species
[e] Direct submission to Genbank.

References

Avila-Adame, C., Gómez-Alpizar, L., Zismann, V., Jones, K.M., Buell, C.R. & Ristaino, J.B. (2006). Mitochondrial genome sequences and molecular evolution of the Irish potato famine pathogen, *Phytophthora infestans*. *Current Genetics* 49: 39-46.

Cai, G. & Scofield, S.R. (2020) Mitochondrial genome sequence of *Phytophthora sansomeana* and comparative analysis of *Phytophthora* mitochondrial genomes. *PLoS ONE*. 15: e0231296

Derevnina, L., Chin-Wo-Reyes, S., Martin, F.N., Wood, K., Froenicke, L., Spring, O. & Michelmore, R. (2015). Genome sequence and architecture of the tobacco downy mildew pathogen *Peronospora tabacina*. *Molecular Plant-Microbe Interactions* 28: 1198-215.

Fletcher, K., Klosterman, S.J., Derevnina, L., Martin, F.N., Bertier, L.D., Koike, S., Reyes-Chin-Wo, S., Mou, B. & Michelmore, R. (2018). Comparative genomics of downy mildews reveals potential adaptations to biotrophy. *BMC Genomics* 19: 851.

Lang, B.F. & Forget, L. (1992). The mitochondrial genome of *Phytophthora infestans*. In *Genetic Maps* (S.J. O'Brien, Ed.). Cold Spring Harbor Laboratory Press, NY. pg 133-135.

Lassiter, E.S., Russ, C., Nusbaum, C., Zeng, Q., Saville, A.C., Olarte, R.A., Carbone, I., Hu, C.-H., Seguin-Orlando, A., Samaniego, J.A., Thorne, J.L. & Ristaino, J.B. (2015). Mitochondrial genome sequences reveal evolutionary relationships of the *Phytophthora* 1c clade species. *Current Genetics* 61: 567-577.

Levesque, C.A., Brouwer, H., Cano, L., Hamilton, J., Holt, C., Huitema, E., Raffaele, S., Robideau, G.P., Thines, M., Win, J., Zerillo, M.M., Beakes, G.W., Boore, J.L., Busam, D., Dumas, B., Ferriera, S., Fuerstenberg, S.I., Gachon, C.M., Gaulin, E., Govers, F., Grenville-Briggs, L., Horner, N., Hostetler, J., Jiang, R.H., Johnson, J., Krajaejun, T., Lin, H., Meijer, H.J., Moore, B., Morris, P., Phuntmart, V., Puiu, D., Shetty, J., Stajich, J.E., Tripathy, S., Wawra, S., van, West, P., Whitty, B.R., Coutinho, P.M., Henrissat, B., Martin, F., Thomas, P.D., Tyler, B.M., De, Vries, R.P., Kamoun, S., Yandell, M., Tisserat, N. & Buell, C.R. (2010). Genome sequence of the necrotrophic plant pathogen *Pythium ultimum* reveals original pathogenicity mechanisms and effector repertoire. *Genome Biology* 11: R73.

Martin, F.N. (2008). Mitochondrial haplotype determination in the oomycete plant pathogen *Phytophthora ramorum*. *Current Genetics* 54: 23-34.

Martin, F.N., Bensasson, D., Tyler, B.M. & Boore, J.L. (2007). Mitochondrial genome sequences and comparative genomics of *Phytophthora ramorum* and *P. sojae*. *Current Genetics* 51: 285-296.

McGowan, J., O'Hanlon, R., Owens, R.A. & Fitzpatrick, D.A. (2020). Comparative genomic and proteomic analyses of three widespread *Phytophthora* species: *Phytophthora chlamydospora*, *Phytophthora gonapodyides* and *Phytophthora pseudosyringae*. *Microorganisms* 8: 653.

Rahman, A., Góngora-Castillo, E., Bowman, M.J., Childs, K.L., Gent, D.H., Martin, F.N. & Quesada-Ocampo, L.M. (2019). Genome sequencing and transcriptome analysis of the hop downy mildew pathogen *Pseudoperonospora humuli* reveal species-specific genes for molecular detection. *Phytopathology* 109:1354-1366.

Winkworth, R.C., Bellgard, S.E., McLenachan, P.A. & Lockhart, P.J. (2021). The mitogenome of *Phytophthora agathidicida*: Evidence for a not so recent arrival of the "kauri killing" *Phytophthora* in New Zealand. *PLoS ONE* 16: e0250422.

Supplementary Table 2. Statistics for the 34 aligned sequence matrices included in phylogenetic analyses

Locus	No. of taxa	Aligned length	No. of sequence positions			Best-fit substitution model
			constant	variable	gapped	
atp1	72	1521	986	535	0	GTR+I+G
atp6	72	720	419	301	0	HKY+I+G
atp8	72	378	190	188	0	GTR+I+G
atp9	72	228	163	65	0	GTR+I+G
cob	72	1137	694	443	0	GTR+I+G
cox1	72	1479	937	542	0	GTR+I+G
cox2	72	762	471	291	33	GTR+I+G
cox3	72	915	488	427	12	GTR+I+G
nad1	72	981	598	383	0	GTR+I+G
nad2	72	1494	819	675	0	GTR+I+G
nad3	72	354	246	108	0	GTR+I+G
nad4	72	1476	935	541	0	GTR+I+G
nad4L	72	303	196	107	0	GTR+I+G
nad5	72	1995	1198	797	9	GTR+I+G
nad6	72	621	338	283	9	GTR+I+G
nad7	72	1179	682	497	0	GTR+I+G
nad9	72	555	287	268	0	GTR+I+G
nad11	72	1986	948	1038	60	GTR+I+G
rpl2	72	807	413	394	54	GTR+I+G
rpl5	72	534	230	304	36	GTR+I+G
rpl6	72	588	202	386	9	GTR+G
rpl14	72	372	213	159	0	GTR+I+G
rpl16	72	405	201	204	0	HKY+I+G
rps2	72	588	241	347	15	GTR+I+G
rps3	72	750	371	379	0	GTR+I+G
rps4	72	462	242	220	0	GTR+I+G
rps7	72	432	248	184	114	HKY+I+G
rps8	72	381	202	179	6	HKY+I+G
rps10	72	327	126	201	84	GTR+G
rps11	72	399	192	207	141	GTR+I+G
rps12	72	381	219	162	0	HKY+I+G
rps13	72	396	203	193	0	HKY+I+G
rps14	72	300	182	118	0	GTR+I+G
rps19	72	237	138	99	27	GTR+I+G
